# The relationship between diabetes and the dementia risk: a meta-analysis

**DOI:** 10.1186/s13098-024-01346-4

**Published:** 2024-05-14

**Authors:** Fang Cao, Fushuang Yang, Jian Li, Wei Guo, Chongheng Zhang, Fa Gao, Xinxin Sun, Yi Zhou, Wenfeng Zhang

**Affiliations:** 1 School of Basic Medical Sciences, Changchun University of Chinese Medicine, Changchun, 130117 China; 2 College of Chinese Medicine, Changchun University of Chinese Medicine, Changchun, 130117 China; 3 Department of Nutrition, Chinese People’s Armed Police Force Medical Characteristic Center, Tianjin, 300162 China; 4 Department of Geriatrics, Baotou Mengshi Hospital of Traditional Chinese Medicine, Baotou, 014000 China

**Keywords:** Diabetes, Dementia, META

## Abstract

**Background:**

The link between diabetes and dementia risk is not well understood. This study evaluates the factors linking diabetes to dementia onset, providing guidance for preventing dementia in diabetic patients.

**Methods:**

This analysis utilized databases such as PubMed, Embase, Web of Science, and the Cochrane Library to review literature from January 31, 2012, to March 5, 2023. Articles were rigorously assessed using specific inclusion and exclusion criteria. The Newcastle-Ottawa Scale (NOS) was used to evaluate the quality of the studies. Data analysis was performed with STATA 15.0.

**Results:**

The study analyzed 15 articles, covering 10,103,868 patients, with 8,821,516 diagnosed with diabetes. The meta-analysis reveals a substantial association between diabetes and an increased risk of dementia [RR: 1.59, 95%CI (1.40–1.80), *P* < 0.01, *I²*=96.4%]. A diabetes duration of less than five years is linked to a higher dementia risk [RR: 1.29, 95%CI (1.20–1.39), *P* < 0.01, *I²*=92.6%]. Additionally, hypoglycemia significantly raises dementia risk [RR: 1.56, 95%CI (1.13–2.16), *P* < 0.01, *I²*=51.5%]. Analyses of blood sugar control, glycated hemoglobin, and fasting blood sugar indicated no significant effects on the onset of dementia.

**Conclusion:**

Diabetes notably increases dementia risk, particularly where diabetes duration is under five years or hypoglycemia is present.

**Registration:**

The research protocol was registered with PROSPERO and assigned the registration number CRD42023394942.

**Supplementary Information:**

The online version contains supplementary material available at 10.1186/s13098-024-01346-4.

## Introduction

Diabetes, a prevalent chronic metabolic disorder, is marked by elevated blood sugar levels from hypoinsulinism or insulin resistance [1]. The International Diabetes Federation (IDF) highlights a significant increase in global diabetes cases—from 463 million in 2019 to nearly 537 million by 2021.The prevalence is projected to rise by 46% by 2045, especially in developing regions like India and China [2, 3]. Chronic high blood sugar may degrade vital organs, including nerves, blood vessels, the heart, kidneys, and the retina, markedly lowering life quality and posing substantial health risks [4]. Experimental research indicates that high blood sugar can trigger reactive oxygen species, leading to neuroinflammation and significant neuronal losses in areas like the hippocampus, thereby impairing cognitive functions and potentially resulting in dementia [5–8]. Systematic reviews suggest that diabetes heightens the risk of progressing from cognitive impairments to dementia [9]. 

Dementia, a severe neurodegenerative condition, drastically affects the ability to conduct daily activities. According to the World Health Organization, over 550,000 people globally are diagnosed with dementia annually, with nearly 100,000 new cases each year; more than 60% of these cases occur in low and middle-income countries [10]. Age is a primary factor in the onset of dementia, and diabetes significantly exacerbates damage to nerves and blood vessels, thus increasing dementia risk. A 2013 meta-analysis by Kapil Gudala et al. revealed that diabetic patients have a 73% higher risk of dementia compared to non-diabetics, including a 56% increased risk of Alzheimer’s Disease (AD) [11]. Subsequent analyses confirm that diabetes doubles the risk of all-cause dementia, elevating the risks for both vascular dementia and AD [12]. Earlier studies have shown similar trends, with diabetic individuals facing up to twice the risk of developing all forms of dementia, including increases in AD and vascular dementia risks [13]. Reviews have consistently indicated that diabetes, along with hypertension, dyslipidemia, and obesity, is a significant predictor of dementia in those aged over 65 [14]. 

To examine if the risk relationship between diabetes and dementia has evolved recently, a comprehensive systematic review and meta-analysis were conducted. This research, spanning studies from 2013 to 2023, builds upon the 2013 meta-analysis by Kapil Gudala et al. Its purpose is to quantify the association between diabetes and dementia, evaluate factors that might influence this relationship, and compare current findings with the 2013 results to identify any changes in the risk associated with diabetes and dementia over the last decade.

## Materials and methods” section

### Literature search

A literature search was conducted across PubMed, Embase, Web of Science, and the Cochrane Library from January 31, 2012, to March 5, 2023. The search focused on combining “Diabetes Mellitus” with “Dementia.” Details on search strategies are available in the supplementary files.

#### Inclusion and exclusion criteria

##### Inclusion criteria

(1) Studies with adult participants. (2) Research examining the relationship between diabetes and dementia risk. (3) Studies employing cohort, cross-sectional, and other observational methodologies.

##### Exclusion criteria

(1) Papers discussing only dementia or diabetes without addressing the risk relationship between the two. (2) Animal studies or cellular research. (3) Reviews, conference articles, guidelines, and other non-original research. (4) Non-English publications. (5) Studies with fewer than 30 participants, due to limited generalizability.

### Data extraction

Data extraction was independently performed by two authors (YFS and GW) who initially screened titles and abstracts to eliminate unsuitable studies. Full texts of potentially relevant studies were then thoroughly reviewed for final inclusion. Any disagreements were resolved by consulting a third author (LJ).

Both YFS and GW separately collected and verified data using a standardized format. Disputed articles were referred to the third author (LJ) for final adjudication. Extracted data included the author, publication years, study designs, locations, sample sizes, demographic details of participants (age, gender, race), follow-up periods, and diagnostic criteria for diabetes and dementia.

### Quality assessment

The Newcastle-Ottawa Scale (NOS) was utilized to evaluate the quality of the included studies. This tool assesses three dimensions across eight criteria, with comparability scoring up to 2 points and the other criteria scoring 1 point each. Studies with scores from 7 to 9 were considered high quality, while those scoring 4 to 6 were deemed moderate quality. Two evaluators (YFS and GW) independently rated each study and cross-checked their assessments. Any discrepancies were resolved through consultation with a third researcher (LJ).

### Data processing and statistical analysis

To assess the impact of diabetes on the risk of developing dementia, this study used diabetes as the primary outcome. Additional measures encompassed duration of diabetes, blood sugar control factors, hypoglycemia, glycated hemoglobin, and fasting blood glucose.

Statistical processing was performed using Stata 15.0 and R 4.2.1. Heterogeneity was assessed quantitatively with Cochran’s Q test and Higgins *I²*, employing a random-effects model for significant heterogeneity (*P* < 0.10 or *I²* > 50%). Otherwise, a fixed-effects model was used. In cases of high heterogeneity, sensitivity and subgroup analyses were conducted to identify sources. Publication bias was visually inspected using funnel plots and statistically tested with Egger’s and Begg’s tests. The trim-and-fill method was applied to adjust for detected publication biases. A significance level of *P* < 0.05 was used to determine statistical relevance of the findings.

## Results

### Literature search results

**Fig. 1:**
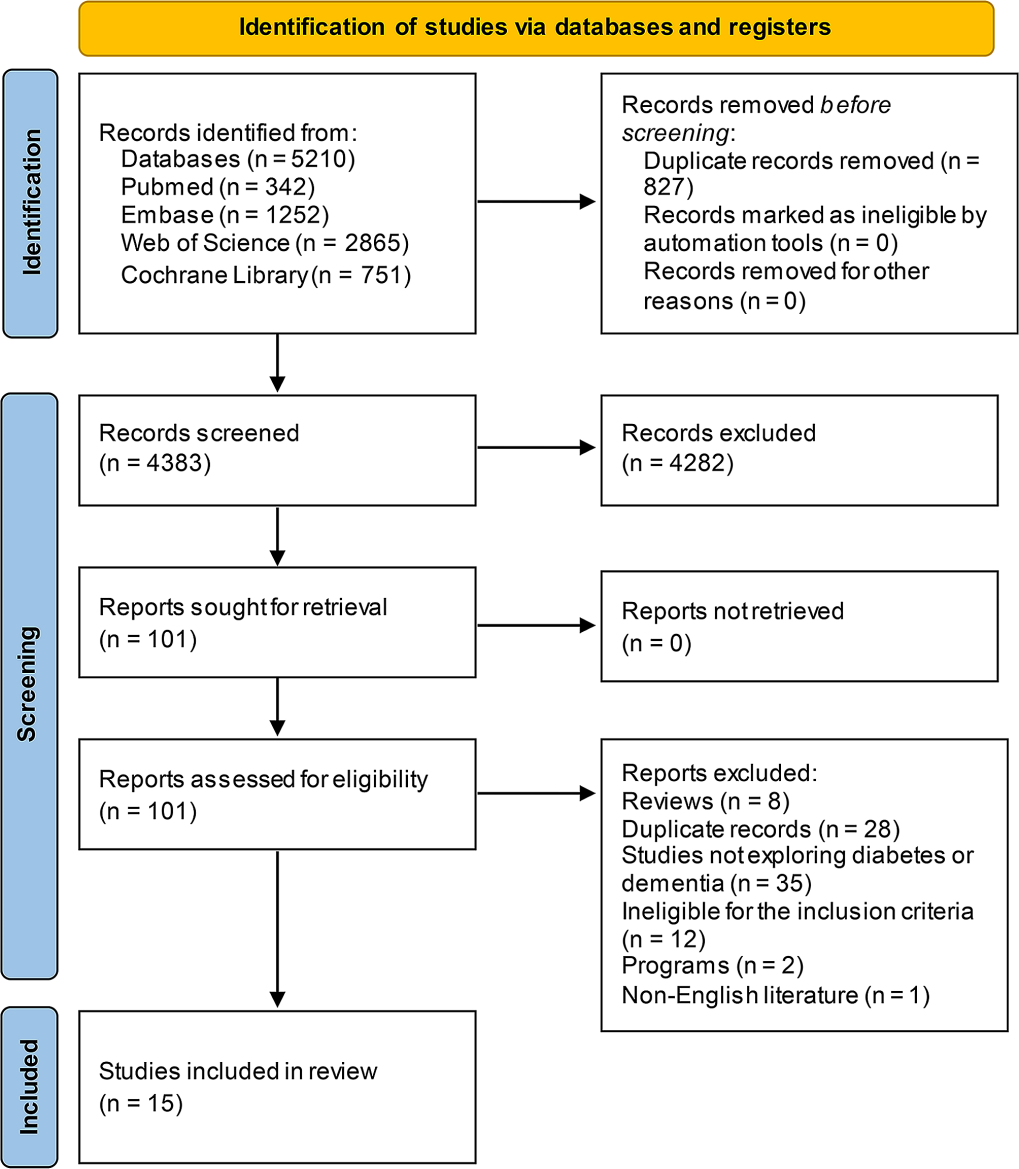
Literature search and selection procedure

A systematic search across PubMed, Embase, Web of Science, and the Cochrane Library yielded 5,210 articles. After removing 827 duplicates, 4,282 articles were excluded during initial screening because their titles and abstracts did not meet the inclusion criteria. This process shortlisted 101 articles for detailed assessment. Upon full-text review, 86 articles were excluded for reasons including irrelevance to the study’s focus on diabetes and dementia, being review articles, or containing duplicate data. Ultimately, 15 articles qualified for inclusion in the analysis. The selection process is depicted in Fig. 1.

### Characteristics of the studies

This analysis included 15 articles [15–29], comprising 12 cohort and three cross-sectional studies. These articles, published between 2012 and 2023, investigated the relationship between diabetes and dementia risk. Most studies focused on Type 2 diabetes, although one specifically addressed Type 1 diabetes [17]. Six studies did not specify diabetes types [16, 18, 21, 22, 25, 28]. Together, these studies accounted for 10,103,868 participants, with 8,821,516 diagnosed with diabetes. Of these, 29,619 cases of dementia were documented, though not all articles provided specific figures for statistical inclusion. Follow-up periods ranged from 0 to 32 years.

Diagnosis of dementia predominantly used International Disease Classification codes (ICD-9, ICD-10), with some studies [18, 27] utilizing the Diagnostic and Statistical Manual of Mental Disorders (DSM-IV) or regional standards like the Korean Classification of Diseases (KCD-6) [20]. Diabetes diagnoses were primarily based on blood sugar levels, although some studies [24] also used patient registration and prescription records. Detailed demographic information and diagnostic criteria are presented in Table 1.

**Table 1:** Descriptive characteristics of the included studies

First author (Publication year)	Study location	Sample size	Gender(male/female)	Age	Type and of diabetics and number of cases	Diagnostic criteria for dementia	Regression method	Follow-up duration(year)	QR
Zheng (2021) [15]	Britain	457,902	238,567/219,335	64.5 ± 10.8	Type 2 diabetes (372,287)		Cox	6 ± 7.5	8
Lee (2022) [16]	Republic of Korea	769,554	468,658/300,896	> 40	Not classified	ICD-10	Cox	6.9 ± 0.815	7
Kuo (2018) [17]	Taiwan, China	33,387	18,229/15,158	48.74 ± 9.79	Type 1 diabetes (1077)	ICD-9	Cox	Inadequate follow-up time	6
Jia (2021) [18]	China	4583	1957/2626	71.32 ± 5	Not classified (658)	DSM-IV	Logistic	Not reported	12
Gennip (2021) [19]	Netherlands	87,856	44,455/43,401	57.1 ± 8.2	Type 2 diabetes (10,663)	ICD-9 ICD-10	Cox	9 ± 0.9	9
Chin (2016) [20]	Republic of Korea	1957	920/1037	67.5 ± 5.5	Type 2 diabetes (1957)	KCD-6	Cox	3.4 ± 0.9	8
Ma (2015) [21]	China	8213	4369/3844	75.81 ± 0.52	Not classified (1134)	DSM-IIIR	Cox	4.17 ± 1.15	8
Frison (2019) [22]	France	8328	3306/5022	73.5 ± 5.778	Not classified (809)	DSM-IV	Cox	8.3 ± 5.481	8
Salinas (2016) [23]	Mexico	1193	415/778	73.2 ± 5.9	Type 2 diabetes (280)	DRG DSM-IV	Poisson	3	7
Secnik (2017) [24]	Sweden	29,630	14,371/15,259	78.8 ± 67.3	Type 2 diabetes (4,881)	ICD-10	Logistic	Not reported	11
Javanshiri (2018) [25]	Sweden	268	113/155	82 ± 16.3	Not classified	the NationalInstitute on Aging /Reagan Institute (1997)		No follow-up	10
Amidei (2021) [26]	Britain	10,095	6794/3301	76.8 ± 6	Type 2 diabetes (1710)	ICD-10	Cox	31.7 ± 1.111	9
MScPH (2021) [27]	Australia	11,140	6406/4734	65.8 ± 6.4	Type 2 diabetes	DSM-IV	logistic	Median: 5	7
Kim (2022) [28]	Republic of Korea	8,400,950	824,028/663,342	< 5, 61.4 ± 10.1; > 5, 64.6 ± 9.1	Not classify Type 1 and Type 2 diabetes, with more cases of Type 2 diabetes	ICD-10	Cox	Average: 5.94	7
Alkabbani (2022) [29]	Canada	278,812	153,235/125,577	40–70	Type 2 diabetes (13,970)	BC health care data, ICD-10 (ICD-9, ICD-10-CA)	Cox	5.03 ± 5.7	8

A quality assessment using the NOS showed that the overall quality of the studies was high, with specific quality scores detailed in Table 1.

### Primary outcome measures and group analysis *relationship between diabetes and dementia onset*

**Fig. 2:**
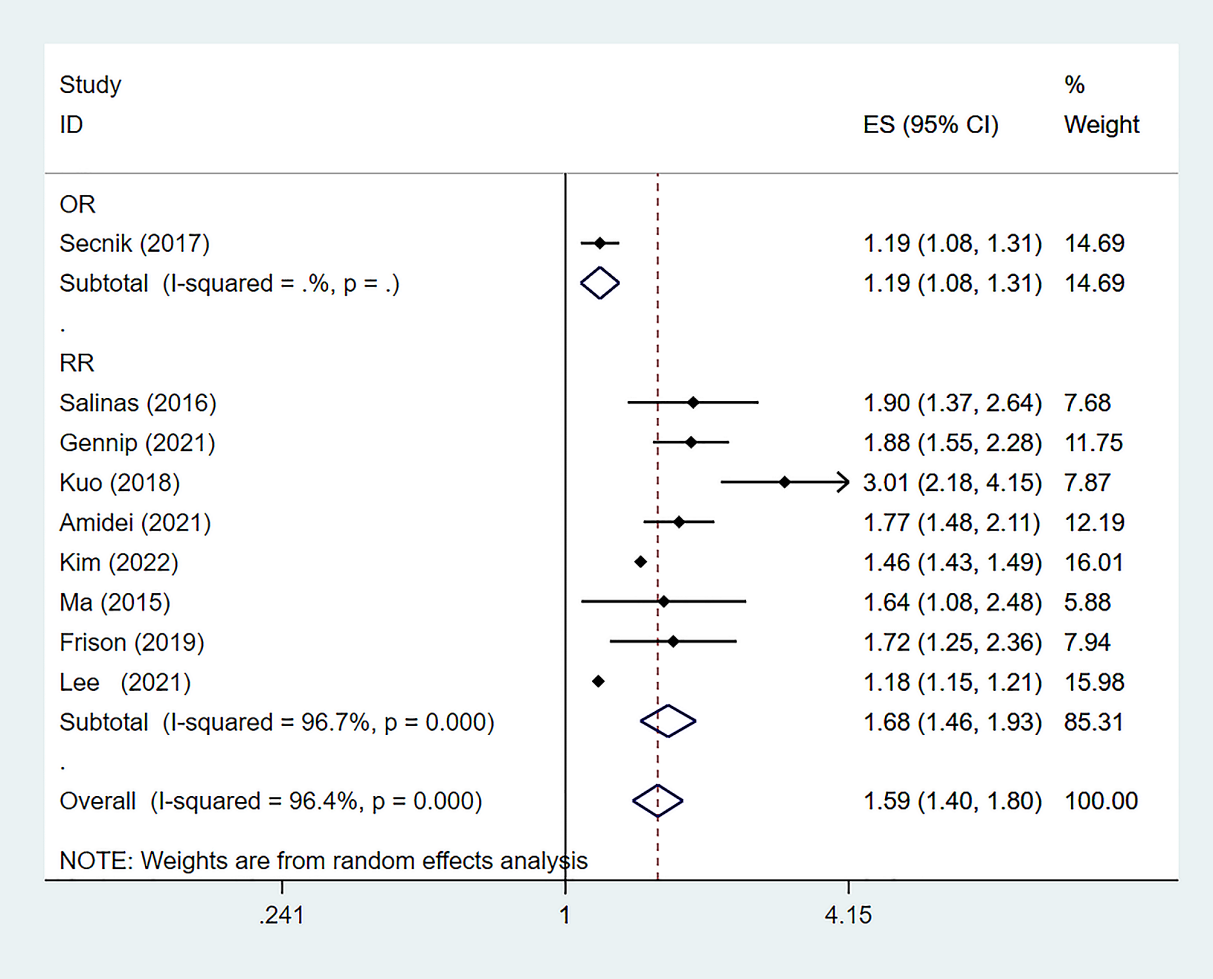
Risk of dementia in diabetes, CI (confidence interval)

**Fig. 3:**
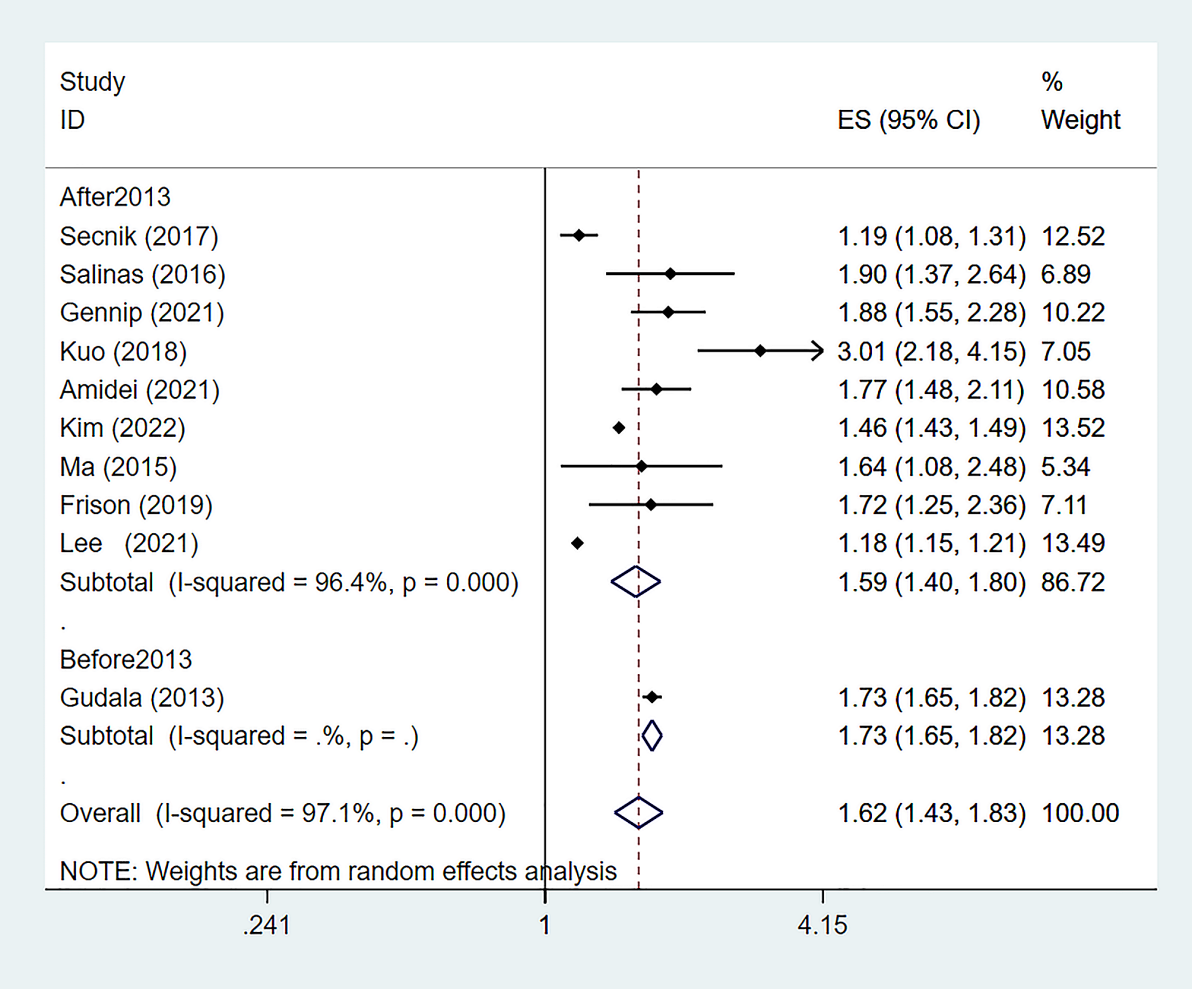
Risk of dementia in diabetes compared with 2013 study data, CI

Among the 15 studies analyzed, 9 [16, 17, 19, 21–24, 26, 28] focused on the relationship between diabetes and dementia onset. These studies showed significant heterogeneity (*P* < 0.01, *I²* = 96.4%), leading to the use of a random-effects model. Results demonstrated a significantly increased risk of dementia in diabetic patients [RR: 1.59, 95%CI (1.40–1.80), *P* < 0.01], as shown in Fig. 2. A comparison with a 2013 meta-analysis, which reported a RR of 1.73 for All Types of Dementia (ATD) in diabetics [95%CI 1.65–1.82, *P* < 0.01], indicated consistent findings. The combined data from the current and 2013 studies reaffirmed diabetes as a significant dementia risk factor [RR: 1.62, 95%CI (1.43–1.83), *P* < 0.01], with persistent high heterogeneity (*P* < 0.01, *I²* = 97.1%), depicted in Fig. 3.

Further analysis assessed the impact of various factors on dementia onset, including diabetes duration in three studies [16, 26, 28], diabetes control in two studies [23, 27], hypoglycemic events in four studies [15, 20, 23, 29], glycosylated hemoglobin in three studies [15, 21, 27], and fasting blood glucose in two studies [23, 26], with details presented in Table 2. Notable heterogeneity was observed in associations for diabetes duration of less than five years (*I²* = 92.6%), hypoglycemic events (*I²* = 51.5%), glycosylated hemoglobin (*I²* = 69.5%), and fasting blood glucose (*I²* = 70.7%), requiring the application of a random-effects model. Conversely, the analysis of diabetes control factors showed minimal heterogeneity (*P* = 0.862, *I²* = 0.0%), allowing for a fixed-effects model.

**Table 2:** Dementia risk associated with other factors

Factors Associated with Dementia Onset Risk	Number of Studies (*n*)	ES (95%CI)	*I*²	*P*
Diabetes duration (< 5 years)	3	1.29 (1.20–1.39)	92.60%	*P* < 0.01
Diabetes control	2	1.00 (0.90–1.11)	0.00%	*P* > 0.05
Hypoglycemic events	4	1.56 (1.13–2.16)	51.50%	*P* < 0.01
Glycated hemoglobin	3	1.04 (1.00-1.09)	69.50%	*P* > 0.05
Fasting blood sugar	2	1.37 (0.78–2.41)	70.70%	*P* > 0.05

Analysis from three articles indicated that shorter durations of diabetes are associated with an increased risk of dementia. Specifically, patients with diabetes for less than five years exhibited a 29% higher risk of dementia compared to their non-diabetic counterparts [RR: 1.29, 95%CI (1.20–1.39), *P* < 0.01], as shown in Supplementary Fig. 1. Four studies highlighted a significant relationship between hypoglycemic events and the onset of dementia. Patients experiencing hypoglycemic events faced a 56% increased risk of dementia [RR: 1.56, 95%CI (1.13–2.16), *P* = 0.007], presented in Supplementary Fig. 2.

The meta-analysis revealed a low correlation between dementia onset and diabetes management factors such as blood glucose control, glycosylated hemoglobin, and fasting blood glucose. No statistically significant differences were observed in dementia onset between patients with and without managed blood sugar levels [RR: 1.00, 95%CI (0.90–1.11), *P* = 0.988], outlined in Supplementary Fig. 3. A minimal association was found between glycosylated hemoglobin levels and increased dementia risk [RR: 1.04, 95%CI (1.00-1.09), *P* = 0.052], as illustrated in Supplementary Fig. 4. Similarly, fasting blood glucose levels suggested a 37% increase in dementia risk, although this was not statistically significant [RR: 1.37, 95%CI (0.78–2.41), *P* = 0.275], as detailed in Supplementary Fig. 5.

### Sensitivity analysis

**Fig. 4:**
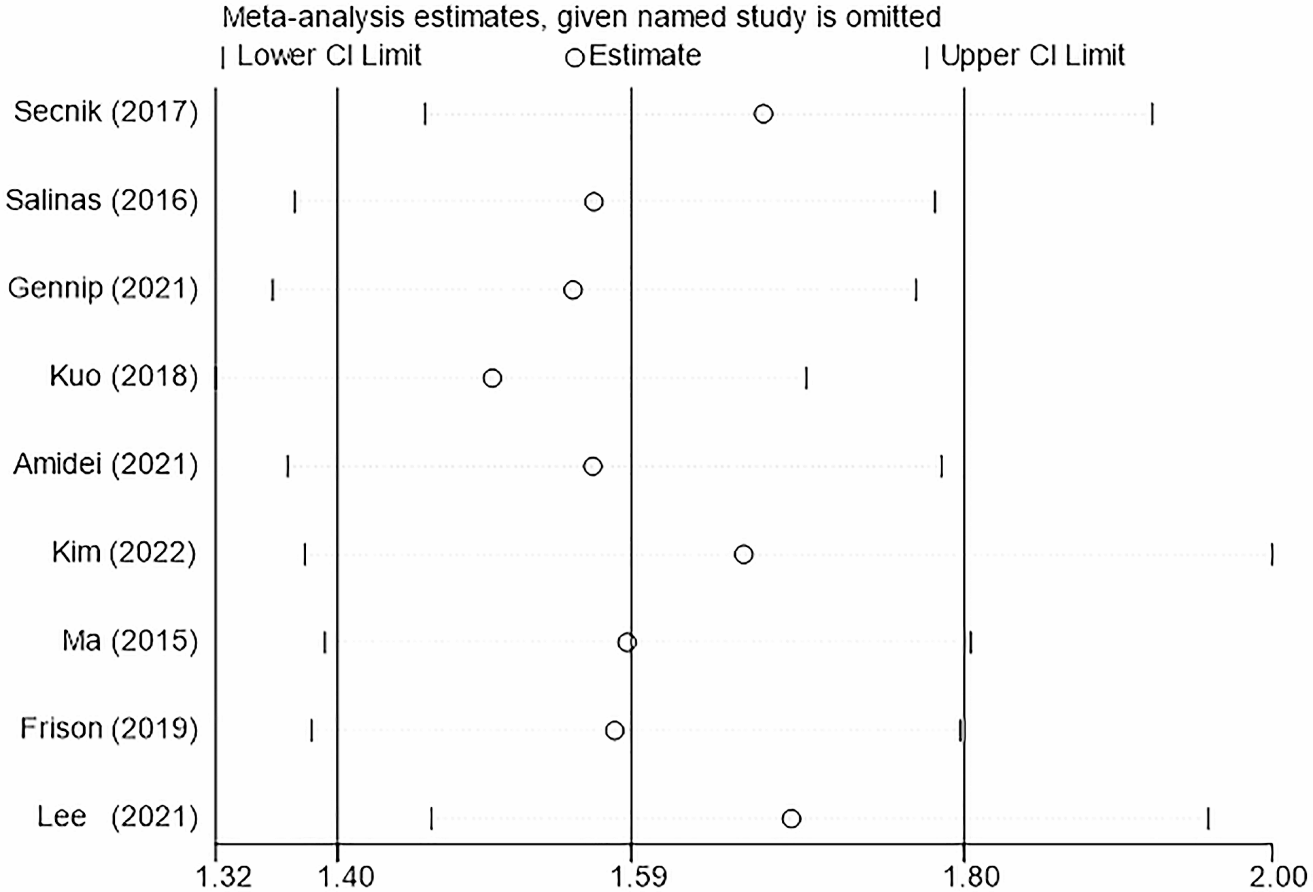
Sensitivity analysis

Sensitivity analysis was conducted to assess the stability of the findings related to the link between diabetes and dementia onset. By sequentially excluding each study, the results consistently fell within the range of the overall effect value, indicating that the conclusions are robust and stable. These details are shown in Fig. 4.

### Publication Bias

Publication bias was assessed visually using a funnel plot and statistically through Begg’s and Egger’s tests. The results of these tests showed values of *P*_Begg_ = 1.00 and *P*_Egger_ = 0.429, both above the threshold of 0.05, suggesting no significant publication bias. This analysis is illustrated in Supplementary Fig. 6.

## Discussion

This meta-analysis evaluated the results of 15 observational studies and discovered that diabetic patients have a 59% increased risk of developing dementia compared to non-diabetics. This finding is consistent with a 2013 meta-analysis (RR: 1.73, 95%CI: 1.65–1.82).

Research has investigated the connection between diabetes and dementia. Leibson and colleagues found in their meta-analysis that among 1,455 adult diabetic patients tracked for 15 years, 101 developed dementia and 77 developed AD, indicating a 66% increased risk [30]. Similarly, Salinas et al. observed that the risk of dementia in Mexican patients with type 2 diabetes was double that of non-diabetics [23]. This aligns with results from a large prospective cohort study in Rotterdam, the Netherlands, which noted that diabetes nearly doubles the risk of dementia and AD [31]. A comprehensive review of 243 prospective studies and 153 randomized control trials identified diabetes as a significant risk factor, increasing the risk of AD by 69% [32]. Additionally, another study indicated that diabetic patients have a 1.25 to 1.91 times higher risk of cognitive impairment and dementia, with increased risks manifesting even in the early stages of diabetes [33]. 

These studies collectively support the robust link between diabetes and the onset of dementia, highlighting that this association may begin as early as within the first five years of diabetes. The underlying mechanisms of this relationship, however, remain to be fully elucidated.

Biological studies have shown that diabetes-related complications such as insulin resistance, acute fluctuations in blood glucose, changes in blood flow rate, and chronic inflammation may increase the risk of dementia. Research involving diabetic animals has demonstrated that diminished granule cell proliferation in the hippocampal areas CA1 and CA3 results in neuronal apoptosis and, consequently, dementia. Diabetes is also associated with brain damage, manifesting as cerebral atrophy, altered brain vasculature, impaired synaptic plasticity, and neuroglial cell dysfunction, all of which compromise brain function [34–36]. 

Imaging and neuropathological studies indicate that the brains of diabetic patients may undergo changes akin to those seen in early-stage AD patients [12, 37]. AD is characterized by the accumulation of amyloid-beta (Aβ) proteins, hyperphosphorylation of Tau proteins, and the formation of neurofibrillary tangles, all contributing to the onset of dementia [38]. 

Recent studies confirm that dementia and cognitive impairments are significant complications associated with Type 2 diabetes (T2D) [39]. With the increasing prevalence of T2D and challenges in self-management among patients, the incidence of dementia is expected to rise [40]. Although the effect of diabetes duration on dementia onset requires further study. The American ARIC study indicated that a longer duration of diabetes correlates with an increased risk of developing dementia [41]. Findings suggest that cognitive function declines sharply within the first few years after diabetes onset, and this decline is not significantly influenced by age [42, 43]. Likely, our research reveals a 29% increased risk of dementia when the duration of diabetes is less than five years, indicating that the impact of diabetes on dementia may begin early in the disease process. This is in line with a UK prospective study, which has identified a clear linear relationship between the duration of diabetes, blood sugar control, and the onset of dementia, showing a 15% increase in dementia incidence for every five years of diabetes duration [44]. Similarly, a German prospective study found higher rates of dementia diagnosis in the early stages of diabetes, consistent with our findings, suggesting that cognitive impairment may start even before diabetes is clinically diagnosed due to prolonged abnormal blood sugar levels [45]. 

Our research indicates that hypoglycemic events significantly escalate the risk of dementia among diabetic patients. Hypoglycemia, a common acute complication of diabetes, can cause substantial vascular damage in the brain, leading to neuronal injury and contributing to cognitive impairments [20, 46, 47]. Cohort studies reveal that diabetic patients with severe hypoglycemia are at an increased risk of developing dementia. Notably, there is a linear relationship between the frequency of severe hypoglycemic episodes and dementia risk, especially among AD patients. Even a single severe episode can heighten the risk of mortality, independent of dementia status [48]. 

Further studies underscore that elderly T2D patients with a history of severe hypoglycemia face a higher dementia risk, suggesting a potential bidirectional relationship between hypoglycemia and dementia risk in this population [49, 50]. Our findings corroborate that diabetic patients experiencing hypoglycemic events have a 56% increased risk of developing dementia compared to those without such episodes, confirming the detrimental impact of hypoglycemia on brain health and consequently dementia.

Additionally, a systematic review has quantified this relationship, noting that severe hypoglycemic events raise the risk of dementia [RR: 1.47, 95%CI (1.24–1.74)]. The risk escalates with the frequency of episodes: one event increases the risk by 29% [RR: 1.29, 95%CI (1.15–1.44)], two episodes by 68% [RR: 1.68, 95%CI (1.38–2.04)], and three or more episodes double the risk [RR: 1.99, 95%CI (1.48–2.68)] [51]. These findings highlight the significant role of hypoglycemic events as a factor in the development of dementia in diabetic patients.

### Strengths and limitations

This meta-analysis encompasses studies from 2012 to 2023, providing an updated and comprehensive evaluation of the relationship between diabetes and dementia. It also examines critical factors such as early-stage diabetes, hypoglycemia, diabetes control, glycated hemoglobin, and fasting blood sugar, enhancing our understanding of the intricate link between diabetes and dementia.

Despite its strengths, this study has several limitations. The relatively small number of included studies may restrict the breadth and representativeness of the patient population. This limitation prevented in-depth subgroup analyses by different dementia types like vascular dementia or AD, or by diabetes types such as Type 1, Type 2, or gestational diabetes, which could influence the outcomes. Additionally, the analysis of diabetes-related factors was limited, and the absence of tiered analysis for these factors may affect the robustness of the results. Excluding studies published in non-English languages may also introduce selection bias.

Our findings reaffirm that diabetic patients have a higher risk of dementia, aligning with conclusions from a 2013 study. Future research should incorporate larger sample sizes and adopt a longitudinal approach to investigate various risk factors more accurately. Further studies are encouraged to include diverse types of dementia and diabetes, expand the number of studies reviewed, increase the data volume, and assess the impact of measurement errors on risk estimates.

### Electronic supplementary material

Below is the link to the electronic supplementary material.

Supplementary Material 1: Search strategy. Figure S1: Association between diabetes duration (<5 years) and risk of dementia; CI, confidence interval. Figure S2: Association between hypoglycemic events and risk of dementia; CI, confidence interval. Figure S3: Association between diabetes control and risk of dementia; CI, confidence interval. Figure S4: Association between glycated hemoglobin and risk of dementia; CI, confidence interval. Figure S5: Association between fasting blood glucose and risk of dementia, CI, confidence interval. Figure S6: Publication bias.

## Data Availability

No datasets were generated or analysed during the current study.

## Abbreviations

The Newcastle: Ottawa Scale (NOS)
AD: Alzheimer’s Disease
GLP-1: Incretin glucagon-like peptide1
SGLT-2: Sodium-glucose cotransporter2
