# Supplementary material for: The relationship between diabetes and the dementia risk: a meta-analysis
Source: Diabetol Metab Syndr. 2024 May 14;16:101. doi: 10.1186/s13098-024-01346-4 (PMC11092065; doi:10.1186/s13098-024-01346-4)
Supplement: Supplementary file 1 — Supplementary Material 1: Search strategy. Figure S1: Association between diabetes duration (<5 years) and risk of dementia; CI, confidence interval. Figure S2: Association between hypoglycemic events and risk of dementia; CI, confidence interval. Figure S3: Association between diabetes control and risk of dementia; CI, confidence interval. Figure S4: Association between glycated hemoglobin and risk of dementia; CI, confidence interval. Figure S5: Association between fasting blood glucose and risk of dementia, CI, confidence interval. Figure S6: Publication bias. [file 13098_2024_1346_MOESM1_ESM.docx]

**Supplementary materials**

**Supplementary file 1:** Search strategy

1. Embase

| No. | Query | Results |
| --- | --- | --- |
| #12 | #8 AND #9 AND #10 AND [2012-2022]/py | 1082 |
| #11 | #8 AND #9 AND #10 | 2178 |
| #10 | #3 OR #7 | 2270230 |
| #9 | #2 OR #5 | 444945 |
| #8 | #1 OR #4 | 1345397 |
| #7 | 'clinical trial':ab,ti OR 'clinical trial as topic':ab,ti OR 'intervention study':ab,ti OR 'clinic trial':ab,ti OR 'controlled clinical trial':ab,ti OR trial:ab,ti OR 'clinical study':ab,ti OR 'clinical trial':it | 1115040 |
| #6 | 'clinical trial':ab,ti OR 'clinical trial as topic':ab,ti OR 'intervention study':ab,ti OR 'clinic trial':ab,ti OR 'controlled clinical trial':ab,ti OR trial:ab,ti OR 'clinical study':ab,ti | 1115040 |
| #5 | dementia:ab,ti OR dementias:ab,ti OR demention:ab,ti OR amentia:ab,ti OR amentias:ab,ti OR 'senile paranoid dementia':ab,ti OR 'dementias, senile paranoid':ab,ti OR 'paranoid dementia, senile':ab,ti OR 'paranoid dementias, senile':ab,ti OR 'senile paranoid dementias':ab,ti OR 'familial dementia':ab,ti OR 'dementia, familial':ab,ti OR 'dementias, familial':ab,ti OR 'familial dementias':ab,ti OR 'alzheimer disease':ab,ti | 200536 |
| #4 | 'diabetes mellitus':ab,ti OR diabetes:ab,ti OR diabetic:ab,ti | 1085892 |
| #3 | 'clinical trial'/exp | 1727960 |
| #2 | 'dementia'/exp | 413129 |
| #1 | 'diabetes mellitus'/exp | 1160920 |

2. PubMed

| Search number | Query | Sort By | Search Details | Results | |  |
| --- | --- | --- | --- | --- | --- | --- |
| 12 | ((("Diabetes Mellitus"[Mesh]) OR (((diabetes mellitus[Title/Abstract]) OR (diabetes[Title/Abstract])) OR (diabetic[Title/Abstract]))) AND (("Dementia"[Mesh]) OR (((((((((((((((Dementia[Title/Abstract]) OR (Dementias[Title/Abstract])) OR (demention[Title/Abstract])) OR (Amentia[Title/Abstract])) OR (Amentias[Title/Abstract])) OR (Senile Paranoid Dementia[Title/Abstract])) OR (Dementias, Senile Paranoid[Title/Abstract])) OR (Paranoid Dementia, Senile[Title/Abstract])) OR (Paranoid Dementias, Senile[Title/Abstract])) OR (Senile Paranoid Dementias[Title/Abstract])) OR (Familial Dementia[Title/Abstract])) OR (Dementia, Familial[Title/Abstract])) OR (Dementias, Familial[Title/Abstract])) OR (Familial Dementias[Title/Abstract])) OR (Alzheimer disease[Title/Abstract])))) AND ((("Clinical Trial" [Publication Type]) OR ("Clinical Trials as Topic"[Mesh])) OR (((((((Clinical Trial[Title/Abstract]) OR (Clinical Trial as Topic[Title/Abstract])) OR (Intervention Study[Title/Abstract])) OR (clinic trial[Title/Abstract])) OR (controlled clinical trial[Title/Abstract])) OR (trial[Title/Abstract])) OR (clinical study[Title/Abstract]))) | | (("Diabetes Mellitus"[MeSH Terms] OR ("Diabetes Mellitus"[Title/Abstract] OR "diabetes"[Title/Abstract] OR "diabetic"[Title/Abstract])) AND ("Dementia"[MeSH Terms] OR ("Dementia"[Title/Abstract] OR "Dementias"[Title/Abstract] OR "demention"[Title/Abstract] OR "Amentia"[Title/Abstract] OR "Amentias"[Title/Abstract] OR (("Senile"[All Fields] OR "seniles"[All Fields] OR "senility"[All Fields]) AND "paranoid dementia"[Title/Abstract]) OR (("Dementia"[MeSH Terms] OR "Dementia"[All Fields] OR "Dementias"[All Fields] OR "dementia s"[All Fields]) AND "senile paranoid"[Title/Abstract]) OR (("Paranoid"[All Fields] OR "paranoids"[All Fields]) AND "dementia senile"[Title/Abstract]) OR ((("Paranoid"[All Fields] OR "paranoids"[All Fields]) AND ("Dementia"[MeSH Terms] OR "Dementia"[All Fields] OR "Dementias"[All Fields] OR "dementia s"[All Fields])) AND "Senile"[Title/Abstract]) OR ((("Senile"[All Fields] OR "seniles"[All Fields] OR "senility"[All Fields]) AND ("Paranoid"[All Fields] OR "paranoids"[All Fields])) AND "Dementias"[Title/Abstract]) OR "familial dementia"[Title/Abstract] OR "dementia familial"[Title/Abstract] OR "dementias familial"[Title/Abstract] OR "familial dementias"[Title/Abstract] OR "alzheimer disease"[Title/Abstract])) AND ("Clinical Trial"[Publication Type] OR "Clinical Trials as Topic"[MeSH Terms] OR ("Clinical Trial"[Title/Abstract] OR "clinical trial as topic"[Title/Abstract] OR "intervention study"[Title/Abstract] OR "clinic trial"[Title/Abstract] OR "controlled clinical trial"[Title/Abstract] OR "Trial"[Title/Abstract] OR "clinical study"[Title/Abstract]))) AND (2012/1/1:2022/7/17[pdat]) | 305 | |  |
| 11 | ((("Diabetes Mellitus"[Mesh]) OR (((diabetes mellitus[Title/Abstract]) OR (diabetes[Title/Abstract])) OR (diabetic[Title/Abstract]))) AND (("Dementia"[Mesh]) OR (((((((((((((((Dementia[Title/Abstract]) OR (Dementias[Title/Abstract])) OR (demention[Title/Abstract])) OR (Amentia[Title/Abstract])) OR (Amentias[Title/Abstract])) OR (Senile Paranoid Dementia[Title/Abstract])) OR (Dementias, Senile Paranoid[Title/Abstract])) OR (Paranoid Dementia, Senile[Title/Abstract])) OR (Paranoid Dementias, Senile[Title/Abstract])) OR (Senile Paranoid Dementias[Title/Abstract])) OR (Familial Dementia[Title/Abstract])) OR (Dementia, Familial[Title/Abstract])) OR (Dementias, Familial[Title/Abstract])) OR (Familial Dementias[Title/Abstract])) OR (Alzheimer disease[Title/Abstract])))) AND ((("Clinical Trial" [Publication Type]) OR ("Clinical Trials as Topic"[Mesh])) OR (((((((Clinical Trial[Title/Abstract]) OR (Clinical Trial as Topic[Title/Abstract])) OR (Intervention Study[Title/Abstract])) OR (clinic trial[Title/Abstract])) OR (controlled clinical trial[Title/Abstract])) OR (trial[Title/Abstract])) OR (clinical study[Title/Abstract]))) | | ("Diabetes Mellitus"[MeSH Terms] OR ("Diabetes Mellitus"[Title/Abstract] OR "diabetes"[Title/Abstract] OR "diabetic"[Title/Abstract])) AND ("Dementia"[MeSH Terms] OR ("Dementia"[Title/Abstract] OR "Dementias"[Title/Abstract] OR "demention"[Title/Abstract] OR "Amentia"[Title/Abstract] OR "Amentias"[Title/Abstract] OR (("Senile"[All Fields] OR "seniles"[All Fields] OR "senility"[All Fields]) AND "paranoid dementia"[Title/Abstract]) OR (("Dementia"[MeSH Terms] OR "Dementia"[All Fields] OR "Dementias"[All Fields] OR "dementia s"[All Fields]) AND "senile paranoid"[Title/Abstract]) OR (("Paranoid"[All Fields] OR "paranoids"[All Fields]) AND "dementia senile"[Title/Abstract]) OR ((("Paranoid"[All Fields] OR "paranoids"[All Fields]) AND ("Dementia"[MeSH Terms] OR "Dementia"[All Fields] OR "Dementias"[All Fields] OR "dementia s"[All Fields])) AND "Senile"[Title/Abstract]) OR ((("Senile"[All Fields] OR "seniles"[All Fields] OR "senility"[All Fields]) AND ("Paranoid"[All Fields] OR "paranoids"[All Fields])) AND "Dementias"[Title/Abstract]) OR "familial dementia"[Title/Abstract] OR "dementia familial"[Title/Abstract] OR "dementias familial"[Title/Abstract] OR "familial dementias"[Title/Abstract] OR "alzheimer disease"[Title/Abstract])) AND ("Clinical Trial"[Publication Type] OR "Clinical Trials as Topic"[MeSH Terms] OR ("Clinical Trial"[Title/Abstract] OR "clinical trial as topic"[Title/Abstract] OR "intervention study"[Title/Abstract] OR "clinic trial"[Title/Abstract] OR "controlled clinical trial"[Title/Abstract] OR "Trial"[Title/Abstract] OR "clinical study"[Title/Abstract])) | | 445 | |
| 10 | (("Clinical Trial" [Publication Type]) OR ("Clinical Trials as Topic"[Mesh])) OR (((((((Clinical Trial[Title/Abstract]) OR (Clinical Trial as Topic[Title/Abstract])) OR (Intervention Study[Title/Abstract])) OR (clinic trial[Title/Abstract])) OR (controlled clinical trial[Title/Abstract])) OR (trial[Title/Abstract])) OR (clinical study[Title/Abstract])) | | "Clinical Trial"[Publication Type] OR "Clinical Trials as Topic"[MeSH Terms] OR "Clinical Trial"[Title/Abstract] OR "clinical trial as topic"[Title/Abstract] OR "intervention study"[Title/Abstract] OR "clinic trial"[Title/Abstract] OR "controlled clinical trial"[Title/Abstract] OR "Trial"[Title/Abstract] OR "clinical study"[Title/Abstract] | | 1,596,733 | |
| 9 | ("Dementia"[Mesh]) OR (((((((((((((((Dementia[Title/Abstract]) OR (Dementias[Title/Abstract])) OR (demention[Title/Abstract])) OR (Amentia[Title/Abstract])) OR (Amentias[Title/Abstract])) OR (Senile Paranoid Dementia[Title/Abstract])) OR (Dementias, Senile Paranoid[Title/Abstract])) OR (Paranoid Dementia, Senile[Title/Abstract])) OR (Paranoid Dementias, Senile[Title/Abstract])) OR (Senile Paranoid Dementias[Title/Abstract])) OR (Familial Dementia[Title/Abstract])) OR (Dementia, Familial[Title/Abstract])) OR (Dementias, Familial[Title/Abstract])) OR (Familial Dementias[Title/Abstract])) OR (Alzheimer disease[Title/Abstract])) | | "Dementia"[MeSH Terms] OR ("Dementia"[Title/Abstract] OR "Dementias"[Title/Abstract] OR "demention"[Title/Abstract] OR "Amentia"[Title/Abstract] OR "Amentias"[Title/Abstract] OR (("Senile"[All Fields] OR "seniles"[All Fields] OR "senility"[All Fields]) AND "paranoid dementia"[Title/Abstract]) OR (("Dementia"[MeSH Terms] OR "Dementia"[All Fields] OR "Dementias"[All Fields] OR "dementia s"[All Fields]) AND "senile paranoid"[Title/Abstract]) OR (("Paranoid"[All Fields] OR "paranoids"[All Fields]) AND "dementia senile"[Title/Abstract]) OR ((("Paranoid"[All Fields] OR "paranoids"[All Fields]) AND ("Dementia"[MeSH Terms] OR "Dementia"[All Fields] OR "Dementias"[All Fields] OR "dementia s"[All Fields])) AND "Senile"[Title/Abstract]) OR ((("Senile"[All Fields] OR "seniles"[All Fields] OR "senility"[All Fields]) AND ("Paranoid"[All Fields] OR "paranoids"[All Fields])) AND "Dementias"[Title/Abstract]) OR "familial dementia"[Title/Abstract] OR "dementia familial"[Title/Abstract] OR "dementias familial"[Title/Abstract] OR "familial dementias"[Title/Abstract] OR "alzheimer disease"[Title/Abstract]) | | 247,284 | |
| 8 | ("Diabetes Mellitus"[Mesh]) OR (((diabetes mellitus[Title/Abstract]) OR (diabetes[Title/Abstract])) OR (diabetic[Title/Abstract])) | | "Diabetes Mellitus"[MeSH Terms] OR "Diabetes Mellitus"[Title/Abstract] OR "diabetes"[Title/Abstract] OR "diabetic"[Title/Abstract] | | 787,655 | |
| 7 | ((((((Clinical Trial[Title/Abstract]) OR (Clinical Trial as Topic[Title/Abstract])) OR (Intervention Study[Title/Abstract])) OR (clinic trial[Title/Abstract])) OR (controlled clinical trial[Title/Abstract])) OR (trial[Title/Abstract])) OR (clinical study[Title/Abstract]) | | "clinical trial"[Title/Abstract] OR "clinical trial as topic"[Title/Abstract] OR "intervention study"[Title/Abstract] OR "clinic trial"[Title/Abstract] OR "controlled clinical trial"[Title/Abstract] OR "Trial"[Title/Abstract] OR "clinical study"[Title/Abstract] | | 778,633 | |
| 6 | ((((((((((((((Dementia[Title/Abstract]) OR (Dementias[Title/Abstract])) OR (demention[Title/Abstract])) OR (Amentia[Title/Abstract])) OR (Amentias[Title/Abstract])) OR (Senile Paranoid Dementia[Title/Abstract])) OR (Dementias, Senile Paranoid[Title/Abstract])) OR (Paranoid Dementia, Senile[Title/Abstract])) OR (Paranoid Dementias, Senile[Title/Abstract])) OR (Senile Paranoid Dementias[Title/Abstract])) OR (Familial Dementia[Title/Abstract])) OR (Dementia, Familial[Title/Abstract])) OR (Dementias, Familial[Title/Abstract])) OR (Familial Dementias[Title/Abstract])) OR (Alzheimer disease[Title/Abstract]) | | "Dementia"[Title/Abstract] OR "Dementias"[Title/Abstract] OR "demention"[Title/Abstract] OR "Amentia"[Title/Abstract] OR "Amentias"[Title/Abstract] OR (("Senile"[All Fields] OR "seniles"[All Fields] OR "senility"[All Fields]) AND "paranoid dementia"[Title/Abstract]) OR (("Dementia"[MeSH Terms] OR "Dementia"[All Fields] OR "Dementias"[All Fields] OR "dementia s"[All Fields]) AND "senile paranoid"[Title/Abstract]) OR (("Paranoid"[All Fields] OR "paranoids"[All Fields]) AND "dementia senile"[Title/Abstract]) OR ((("Paranoid"[All Fields] OR "paranoids"[All Fields]) AND ("Dementia"[MeSH Terms] OR "Dementia"[All Fields] OR "Dementias"[All Fields] OR "dementia s"[All Fields])) AND "Senile"[Title/Abstract]) OR ((("Senile"[All Fields] OR "seniles"[All Fields] OR "senility"[All Fields]) AND ("Paranoid"[All Fields] OR "paranoids"[All Fields])) AND "Dementias"[Title/Abstract]) OR "familial dementia"[Title/Abstract] OR "dementia familial"[Title/Abstract] OR "dementias familial"[Title/Abstract] OR "familial dementias"[Title/Abstract] OR "alzheimer disease"[Title/Abstract] | | 145,515 | |
| 5 | ((diabetes mellitus[Title/Abstract]) OR (diabetes[Title/Abstract])) OR (diabetic[Title/Abstract]) | | "diabetes mellitus"[Title/Abstract] OR "diabetes"[Title/Abstract] OR "diabetic"[Title/Abstract] | | 721,993 | |
| 4 | "Clinical Trials as Topic"[Mesh] | Most Recent | "Clinical Trials as Topic"[MeSH Terms] | | 375,262 | |
| 3 | "Clinical Trial" [Publication Type] | Most Recent | "Clinical Trial"[Publication Type] | | 945,516 | |
| 2 | "Dementia"[Mesh] | Most Recent | "Dementia"[MeSH Terms] | | 193,167 | |
| 1 | "Diabetes Mellitus"[Mesh] | Most Recent | "Diabetes Mellitus"[MeSH Terms] | | 483,039 | |

3. Web of Science

| 1 | (diabetes mellitus) OR TS=(diabetes) OR TS=(diabetic) | 860958 |
| --- | --- | --- |
| 2 | TS=(Dementia) OR TS=(Dementias) OR TS=(demention) OR TS=(Amentia) OR TS=(Amentias) OR TS=(Senile Paranoid Dementia) OR TS=(Dementias, Senile Paranoid) OR TS=(Paranoid Dementia, Senile) OR TS=(Paranoid Dementias, Senile) OR TS=(Senile Paranoid Dementias) OR TS=(Familial Dementia) OR TS=(Dementia, Familial) OR TS=(Dementias, Familial) OR TS=(Familial Dementias) OR TS=(Alzheimer disease) | 313381 |
| 3 | TS=(Clinical Trial) OR TS=(Clinical Trials as Topic) OR TS=(Intervention Study) OR TS=(clinic trial) OR TS=(controlled clinical trial) OR TS=(trial) OR TS=(clinical study) | 3941791 |
| 4 | #3 AND #4 AND #5 | 3240 |
| 5 | #3 AND #4 AND #5 2012-01-01/2022-07-18 | 2462 |
|  |  |  |
| 1 | Diabetes Mellitus (Topic) or diabetes (Topic) or diabetic (Topic) | 893,584 |
| 2 | Dementia (Topic) or Dementias (Topic) or demention (Topic) or Amentia (Topic) or Amentias (Topic) or Senile Paranoid Dementia (Topic) or Dementias, Senile Paranoid (Topic) or Paranoid Dementia, Senile (Topic) or Paranoid Dementias, Senile (Topic) or Senile Paranoid Dementias (Topic) or Familial Dementia (Topic) or Dementia, Familial (Topic) or Dementias, Familial (Topic) or Familial Dementias (Topic) or Alzheimer disease (Topic) | 327,935 |
| 3 | Clinical Trial (Topic) or Clinical Trial as Topic (Topic) or Intervention Study (Topic) or clinic trial (Topic) or controlled clinical trial (Topic) or trial (Topic) or clinical study (Topic) | 4,123,955 |
|  | #34 AND #35 AND #39 | [3,444](https://www.webofscience.com/wos/woscc/summary/d0da0f4e-cf3d-47b8-8ca8-21eefd319d88-75be58a2/relevance/1) |
|  | #34 AND #35 AND #39 2022-07-18/2023-03-05 | [223](https://www.webofscience.com/wos/woscc/summary/3a21b1e6-077b-4e88-8297-2268b1c49b22-75be6a63/relevance/1) |


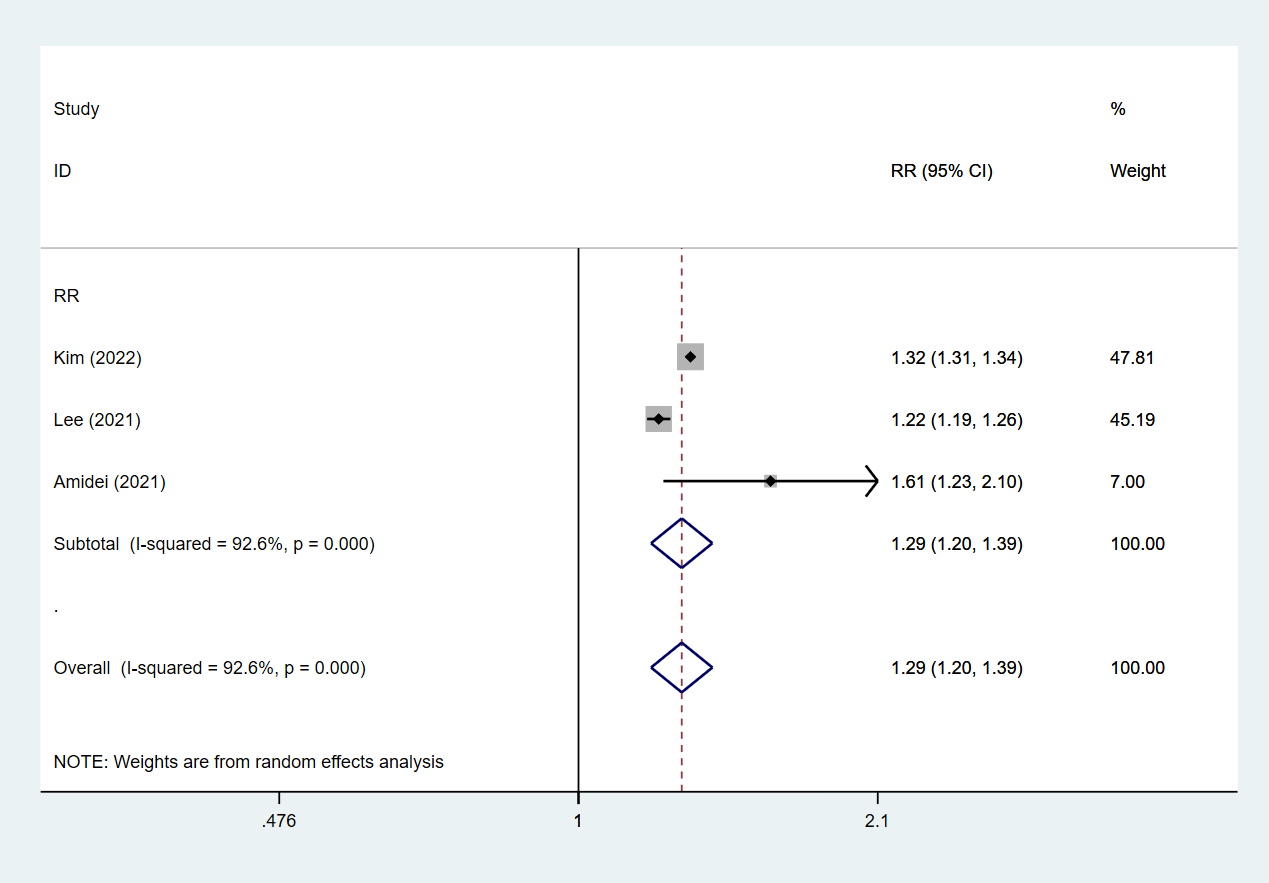


**Figure S1:** Association between diabetes duration (<5 years) and risk of dementia; CI, confidence interval


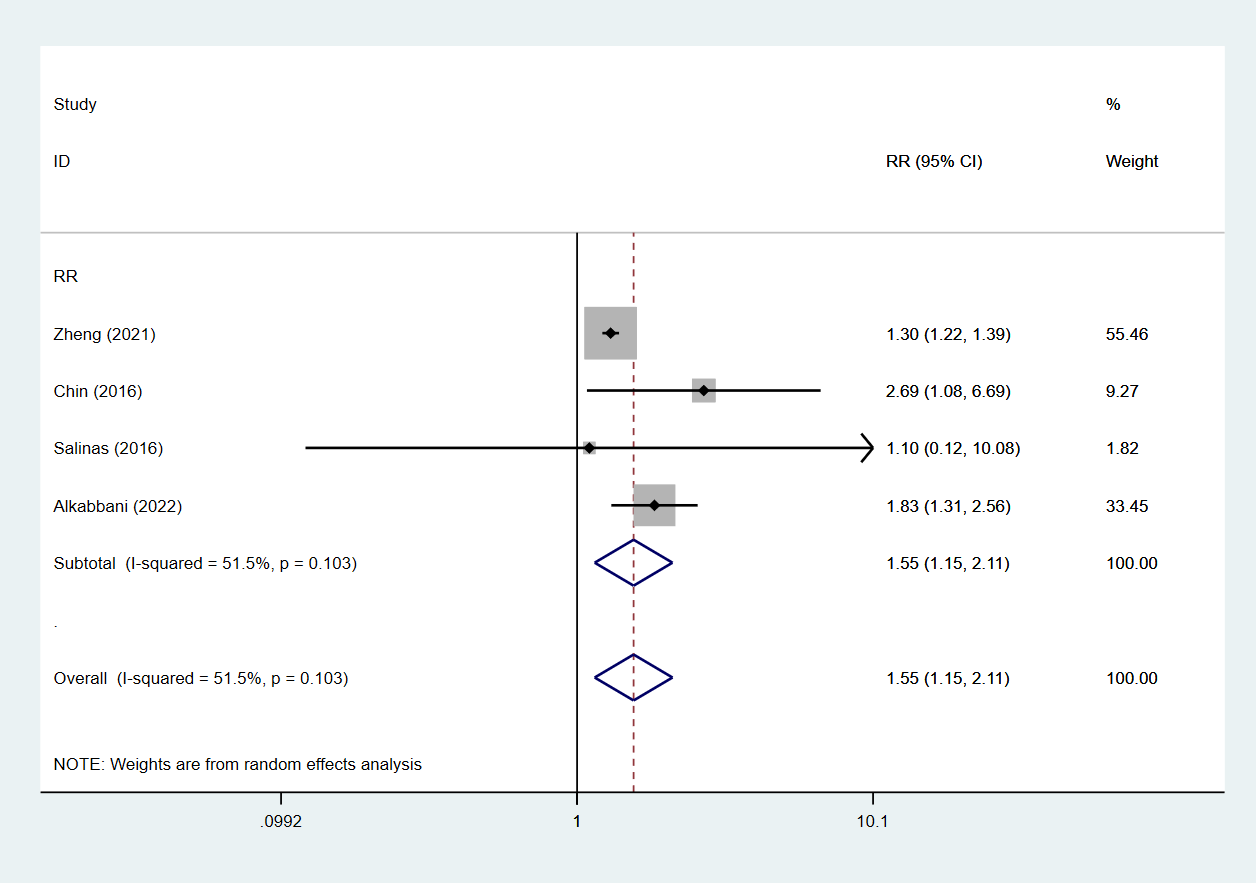


**Figure S2:** Association between hypoglycemic events and risk of dementia; CI, confidence interval


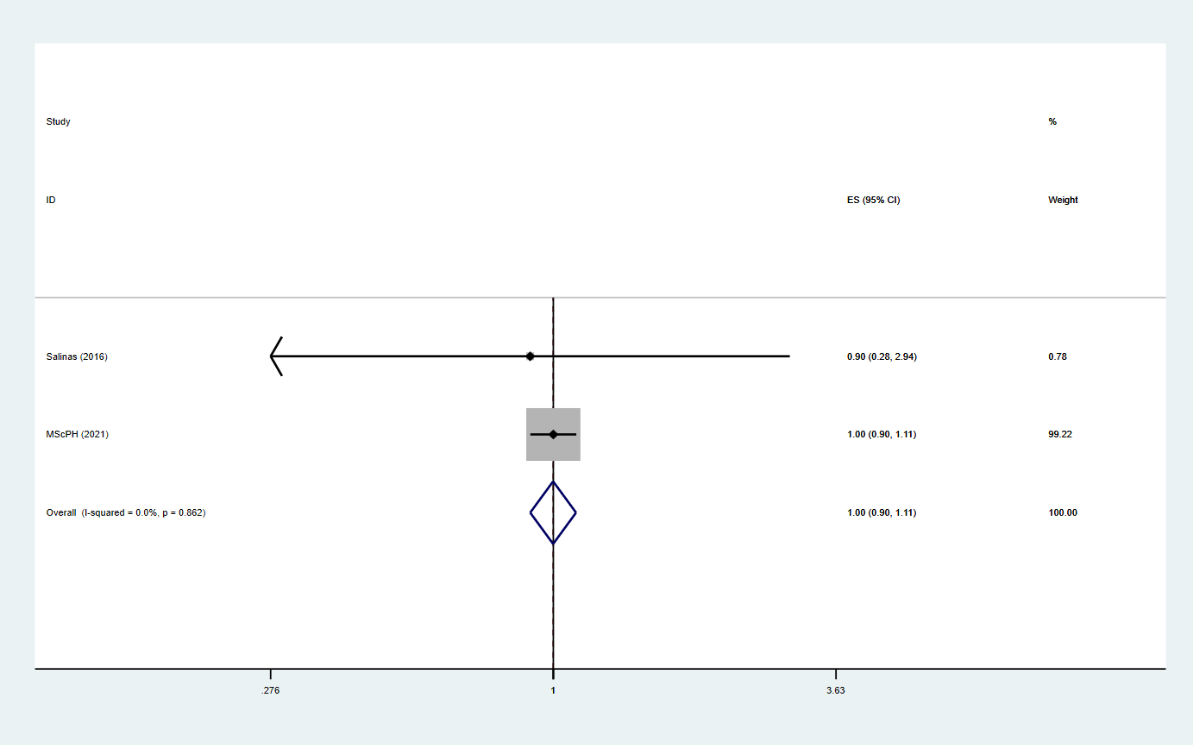
**Figure S3:** Association between diabetes control and risk of dementia; CI, confidence interval


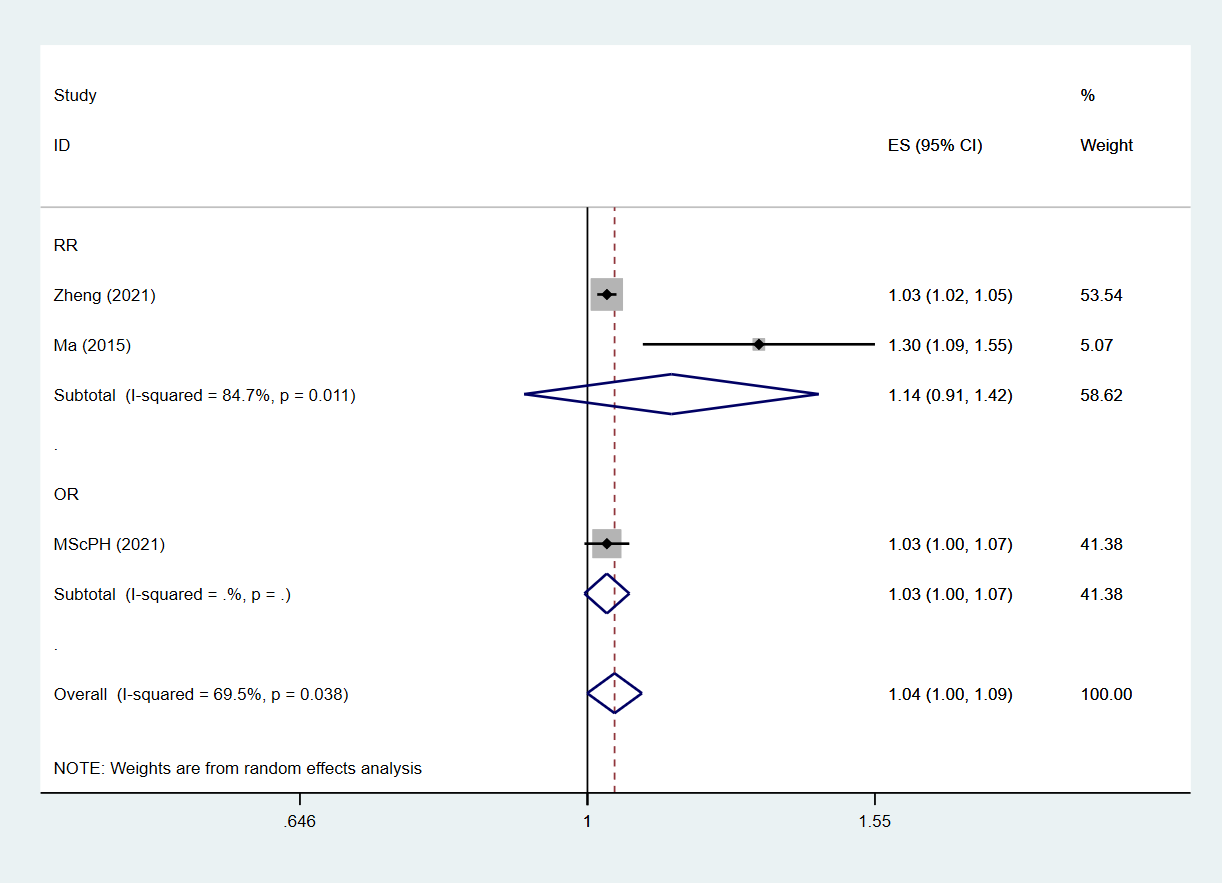


**Figure S4:** Association between glycated hemoglobin and risk of dementia; CI, confidence interval


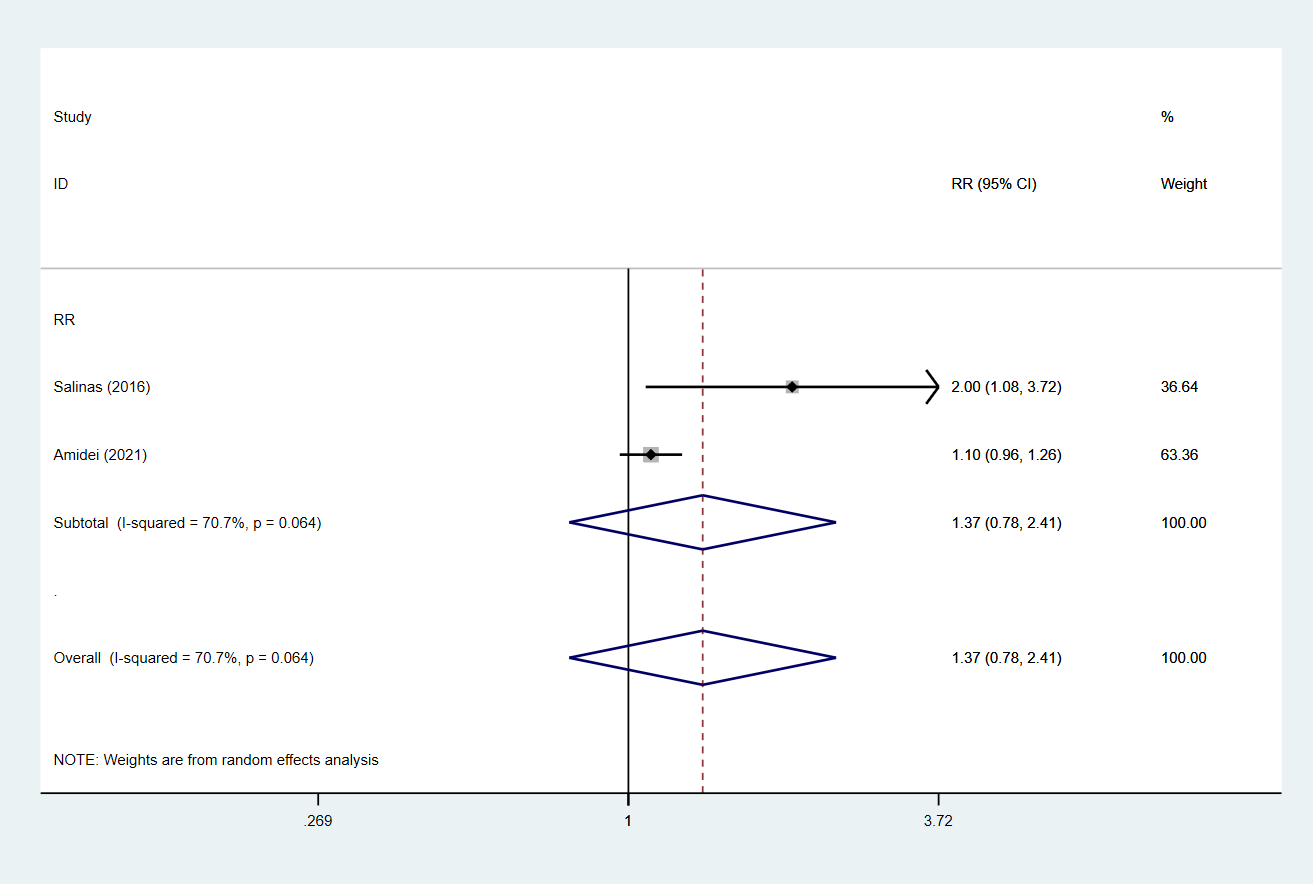
**Figure S5:** Association between fasting blood glucose and risk of dementia, CI, confidence interval

**
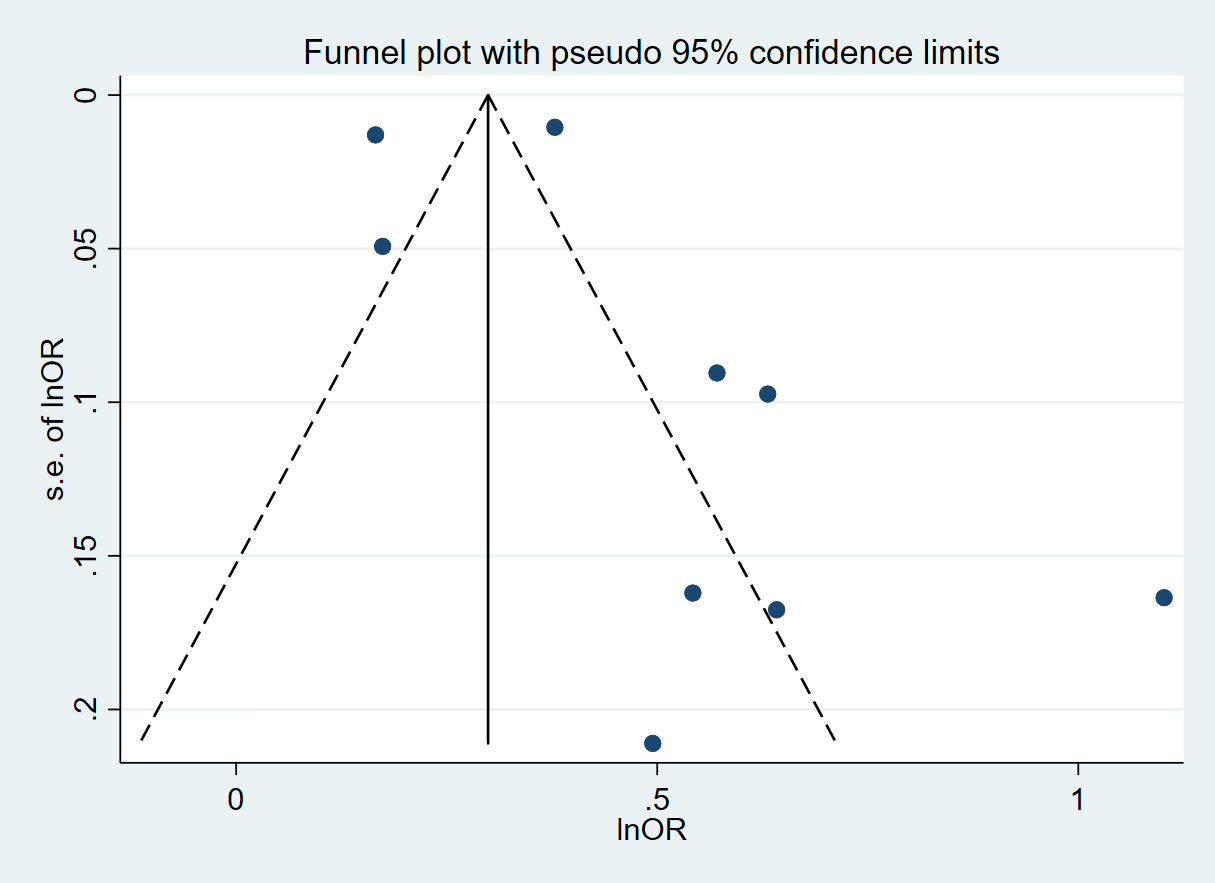
**

**Figure S6:** Publication bias
